# Facile Synthesis Hyper-Crosslinked PdFe Bimetallic Polymer as Highly Active Catalyst for Ullmann Coupling Reaction of Chlorobenzene

**DOI:** 10.3390/polym15122748

**Published:** 2023-06-20

**Authors:** Cheng Tang, Wenwen Yang, Zhijuan Zou, Fang Liao, Chunmei Zeng, Kunpeng Song

**Affiliations:** 1 Chemical Synthesis and Pollution Control Key Laboratory of Sichuan Province, College of Chemistry and Chemical Engineering, China West Normal University, Shida Road, Nanchong 637009, China; 20200043@sasu.edu.cn (C.T.); liaofang407@163.com (F.L.); meizeng@163.com (C.Z.); 2 Key Laboratory of Low-Cost Rural Environmental Treatment Technology, Sichuan University of Arts and Science, Education Department of Sichuan Province, Dazhou 635000, China

**Keywords:** hyper-crosslinked polymer, PdFe nanoparticles, high stability, hydrogenation, Ullmann coupling reaction

## Abstract

The synthesis of efficient and sustainable heterogeneous Pd-based catalysts has been an active field of research due to their crucial role in carbon–carbon coupling reactions. In this study, we developed a facile and eco-friendly in situ assembly technique to produce a PdFe bimetallic hyper-crosslinked polymer (HCP@Pd/Fe) to use as a highly active and durable catalyst in the Ullmann reaction. The HCP@Pd/Fe catalyst exhibits a hierarchical pore structure, high specific surface area, and uniform distribution of active sites, which promote catalytic activity and stability. Under mild conditions, the HCP@Pd/Fe catalyst is capable of efficiently catalyzing the Ullmann reaction of aryl chlorides in aqueous media. The exceptional catalytic performance of HCP@Pd/Fe is attributed to its robust absorption capability, high dispersion, and strong interaction between Fe and Pd, as confirmed by various material characterizations and control experiments. Furthermore, the coated structure of a hyper-crosslinked polymer enables easy recycling and reuse of the catalyst for at least 10 cycles without any significant loss of activity.

## 1. Introduction

Organic synthesis heavily relies on carbon–carbon coupling reactions as a versatile tool to produce complex structures from easily accessible building blocks in diverse ways [1,2,3]. While significant progress has been made in transition metal-catalyzed cross-coupling reactions between aryl halides and organometallic reagents, such as Suzuki, Stille, Negishi, and Hiyama coupling reactions, in the past few decades [4,5,6,7], Ullmann biaryl synthesis remains a straightforward method because of its effectiveness and minimization of undesirable by-products, facilitating the synthesis of various biaryl groups from aryl halides, without the requirement of preformed organometallic species [8,9,10,11,12]. However, the traditional Ullmann coupling reaction has been carried out under harsh reaction conditions, such as high temperature, extended reaction times, use of toxic solvents, and strong bases [13,14]. Despite extensive research on Ullmann coupling reactions, there remains a significant scope to explore recoverable catalysts, mild reaction conditions, and a wider substrate range in this field [15,16,17].

Metal-mediated reactions remain the most widely used approach for constructing aryl–aryl bonds. However, these reactions often require high temperatures (above 100 °C), resulting in energy consumption and environmental concerns [18]. The challenge is further compounded when coupling reactions involve aryl chlorides owing to their higher dissociation energy compared to C–Br or C–I bonds. Despite attempts to use single metallic catalysts to promote the Ullmann coupling reaction of chloroarenes under ambient conditions, these attempts have largely been unsuccessful [19,20]. Recently, Hajipour et al. reported on the successful use of an ortho-palladated complex as a catalyst in the microwave-assisted reaction of different aryl halides (including chloroarenes) at 120 °C. The developed catalyst demonstrated good conversion rates of aryl halides to biaryls in short reaction times, while being insensitive to oxygen. Similarly, another palladacycle has been used to catalyze the homocoupling of aryl halides under microwave irradiation at a reaction temperature of 130 °C, with most of them affording moderate to excellent yields of biaryls when K_2_CO_3_ was used as the base and N-methyl-2-pyrrolidone (NMP) as the solvent. Despite these advances, most solutions still require higher temperatures and microwave conditions with higher energy consumption [21,22]. Recent studies have shown that bimetallic catalysts (such as Pd–Au and Pd–Fe) tend to have better catalytic performances than single metals due to their coordinated effect, making their development a current trend [18].

Hyper-crosslinked polymers (HCPs) are a promising class of organic porous materials that possess remarkable features, such as high porosity, large surface area, well-defined pore architecture, and tunable chemical composition. As a result, HCPs are excellent candidates for various applications, such as gas storage [23,24], heterogeneous catalysis [25,26,27], and others [28,29,30,31]. In particular, HCPs show potential in the field of metal catalysis, as they can provide well-defined microenvironments for incorporating metal nanoparticles owing to their abundant pore structure, which promotes stable metal particles [32,33,34]. In recent years, significant efforts have been devoted to the design and fabrication of metal nanoparticles supported microporous organic polymers (MNP/MOPs) composites. The use of MOPs as host matrices is beneficial not only because of their ability to prevent MNP migration and aggregation but also due to the host–guest synergistic effect that enhances catalytic activity [29]. Recent studies have shown that the bulk electron-rich phosphine can effectively modify the electronic and spatial properties of palladium, promoting cross-coupling reactions [35]. Additionally, the porous structure of HCPs ensures the accessibility of the catalyst and facilitates the transportation and enrichment of substrates to the MNP active sites [36].

For most metal-containing porous polymers (MCPPs), the ligand/metal complex is present on the surface of the supports, resulting in increased cost and difficulty of immobilization as well as the potential leaching of the active site during the reaction process [25,28]. Recently, a simple approach has been proposed to overcome these limitations by knitting rigid aromatic ligands through the Friedel–Crafts reaction to obtain supported catalysts [37,38]. In the field of green chemistry, the development of one-pot multistep transformations is of great interest in recent catalytic chemistry. These transformations have several advantages, such as the reduction of waste, energy, cost, and time by decreasing the number of synthetic steps, thereby conserving chemicals and improving the atomic economy [39].

**Scheme 1:**
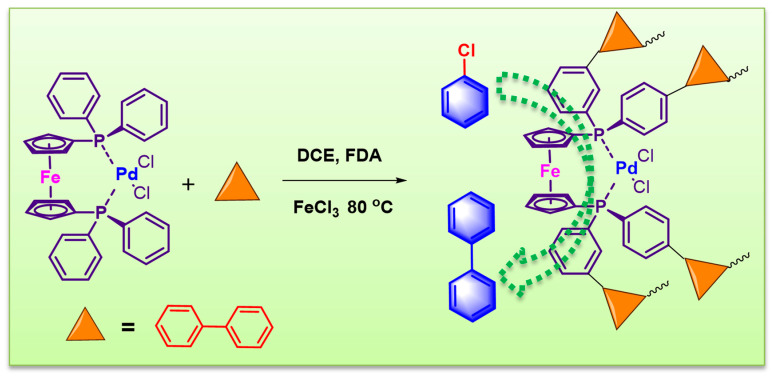
Schematic diagram of the synthetic route of HCP@Pd/Fe.

This study presents a one-pot method for immobilizing the Pd–Fe alloy particles in porous hyper-crosslinked polymers (HCPs) through the in situ knitting of metal-containing ligands (Scheme 1). The resulting catalyst exhibits exceptional performance in Ullmann-coupled reactions with various aryl chlorides, due to its unique coated structure and metal-metal interaction, as the catalyst requires only 0.1 mol% Pd to achieve a 99% yield in the Ullmann reaction, and its TOF reaches up to 376 h^−1^ under the mild reaction conditions. Moreover, the catalyst can be reused for up to 10 cycles without significant loss of activity. The authors suggest that the Pd–Fe metal–metal interaction may enhance the activity and stability of the coupling reaction during the cross-linking process and that this method of constructing low-metal content catalysts through HCPs may provide a promising strategy for achieving efficient and environmentally-friendly C–C Ullmann coupling reactions.

## 2. Experimental Section

### 2.1. Materials

[Bis(diphenylphosphino)ferrocene]dichloropalladium (DPPF-Pd), biphenyl, FeCl_3_, Dichlorobis(Triphenylphosphine)Palladium (PPh_3_-Pd), formaldehyde dimethyl acetal (FDA), Bis(diphenylphosphino)ferrocene] (DPPF), 1,1-dichloroethane (DCE), methanol, 4-nitrophenol (4-NP), NaBH_4_, and other chemical reagents were purchased through Sinopharmatic Reagent Co., LTD (Shanghai, China).

### 2.2. Synthesis of HCP@Pd/Fe

In this study, DPPF-Pd (0.5 mmol) was dissolved in 5 mL of dichloromethane (DCM) with biphenyl (3 mmol) and FDA (6 mmol). Next, FeCl_3_ (12 mmol) was added under vigorous stirring conditions. The reaction mixture was heated to 45 °C for 5 h, to achieve the initial network structure, and then, refluxed at 80 °C for 19 h. The resulting solid products were subjected to Soxhlet Extraction (methanol) at 100 °C until the extracted liquid became colorless. The solid was then vacuum-dried at 80 °C for 24 h, and the sample was identified as HCPs@Pd/Fe. HCPs@Fe was synthesized using DPPF instead of DPPF-Pd, while HCPs@Pd was prepared with PPh_3_-Pd instead of DPPF-Pd, respectively.

### 2.3. Characterization

Fourier-transform infrared (FT-IR) spectra were acquired using the KBr disk method on a Bruker Vertex 70 Spectrometer. Scanning electron microscopy (SEM) images and energy dispersive spectrometer (EDS) element mapping were obtained on a FEI Sirion 200 field emission scanning electron microscope at an accelerating voltage of 10 kV. High-resolution transmission electron microscopy (HR-TEM) images were captured using a Tecnai G2 F30 microscope operated at an accelerating voltage of 200 kV. Atomic absorption spectroscopy (AAS) was utilized to determine the metal content using a Perkin Elmer AA-800 instrument. X-ray photoelectron spectroscopy (XPS) data were collected using a Krato AXIS-ULTRA DLD-600 photoelectron spectrograph. The surface areas of polymer, N_2_ adsorption isotherms (77.3 K), and pore size distributions were measured using Micromeritics ASAP 2020 M surface area and porosity analyzer, after degassing the samples at 110 °C for 8 h under a vacuum.

### 2.4. General Procedure for the Ullmann Reaction

The Ullmann coupling reaction: In the Ullmann reaction protocol, chlorobenzene (1 mmol) and K_3_PO_4_∙3H_2_O (1.5 mmol) were dissolved in a methanol solution (2 mL, *V*_MeOH_/*V*_H2O_ = 1:1) in a 10 mL round bottom flask fitted with a reflux condenser. Then, the catalyst was introduced to the liquid mixture, and the resulting reaction was carried out at 80 °C for 3 h. Following the reaction, the mixture was subjected to centrifugal filtration to isolate the liquid phase, and the yield was analyzed via gas chromatography with a capillary column. For recycling experiments, a 30 mg catalyst was employed. The solid materials were washed with distilled water and ethanol at least three times, dried under a vacuum, and reused in the subsequent run.

## 3. Results and Discussion

### 3.1. Fabrication and Characterization of HCP@Pd/Fe

The HCP@Pd/Fe catalyst was analyzed using Fourier-transform infrared spectroscopy (FT-IR) to investigate its structural characteristics. The FT-IR spectrum (Figure S1) revealed the symmetric and antisymmetric stretching vibration peaks of methylene at 2894 cm^−1^ and 2927 cm^−1^, respectively. Furthermore, the spectrum exhibited a series of bands at 1606–1288 cm^−1^, which can be attributed to the stretching vibration of the benzene ring skeleton. The in-plane and out-of-plane bending vibration of the C–H bond of the benzene ring were detected at 1290–910 cm^−1^ and 860–570 cm^−1^, respectively. These results demonstrate the successful synthesis of the HCP@Pd/Fe catalyst.

**Figure 1:**
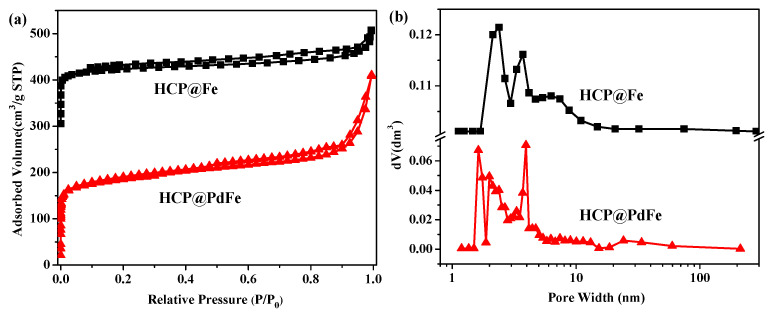
The nitrogen adsorption/desorption isotherm (**a**) and the pore size distribution (**b**) of HCP@Pd/Fe.

The catalysts’ surface area and pore structure were examined by conducting nitrogen adsorption/desorption isotherm measurements at 77 K and the results are shown in Figure 1. The adsorption isotherm curves of HCP@Pd/Fe and HCP@Fe show a steep increase at low relative pressure (*P*/*P*_0_ < 0.01), indicating the presence of abundant micropores, as shown in Figure 1a [34]. HCP@Pd/Fe, synthesized using hyper-crosslinking polymerization, exhibited a higher BET-surface area (402.8 m^2^·g^−1^) than HCP@Fe (384 m^2^·g^−1^), which may be attributed to its larger proportion of micropores (as shown in Figure 1b). Notably, the hysteresis loop and rapid increase in N_2_ adsorption for HCP@Pd/Fe and HCP@Fe were observed at medium relative pressures (*P/P*_0_ = 0.7–0.9), suggesting the presence of mesopores within the highly aggregated particles. Additionally, the presence of microporous structures was inferred from the high-pressure regime of Figure 1a, which is consistent with the results in Figure 1b. The catalyst’s abundant multistage pore structure is likely to facilitate mass transfer during catalytic reactions, thereby enhancing catalytic activity.

**Figure 2:**
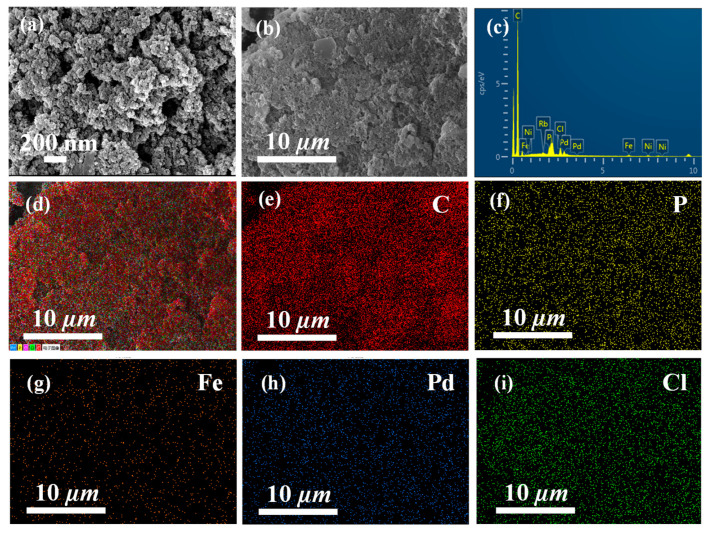
SEM (**a**,**b**), SEM-EDS (**c**,**d**) and elemental mapping images of C (**e**), P (**f**), Fe (**g**), Pd (**h**), Cl (**i**) for the catalyst HCP@Pd/Fe.

The morphology of HCP@Pd/Fe was analyzed through SEM and TEM characterization and revealed a rough and porous surface with stacked holes and a uniform pore distribution (Figure 2a,b). The energy dispersive X-ray spectroscopy (EDS) of HCP@Pd/Fe confirmed the coexistence of C, P, Fe, Pd, and Cl, which were also uniformly distributed throughout the material, as shown in the corresponding SEM-EDS elemental mapping (Figure 2b–i). In order to confirm the arrangement of the two metals in the materials, TEM images were obtained.

**Figure 3:**
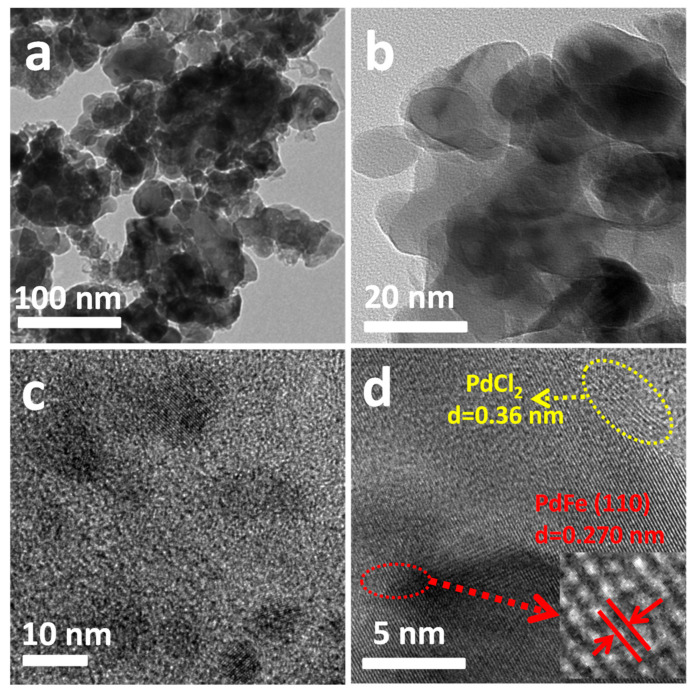
TEM images at different magnifications of HCP@Pd/Fe, (**a**) 100 nm, (**b**) 20 nm, (**c**) 10 nm and (**d**) 5 nm, the illustration in Figure 3d shows the spacing of PdFe alloy crystal faces.

The HCP@Pd/Fe showed a uniform ellipsoidal structure, which was likely formed by conjugate aromatic rings of DPPF-Pd (Figure 3a,b). High-resolution TEM images indicated the presence of a micro-channel, and the metal particles were estimated to be approximately 10 nm in size (Figure 3c). The metal lattice stripes observed were attributed to the crystal plane of PdCl_2_ (0.36 nm) and PdFe alloy (0.27 nm) (Figure 3d), indicating that this work successfully prepared metal alloy active sites through a one-pot hyper-crosslinking method.

**Figure 4:**
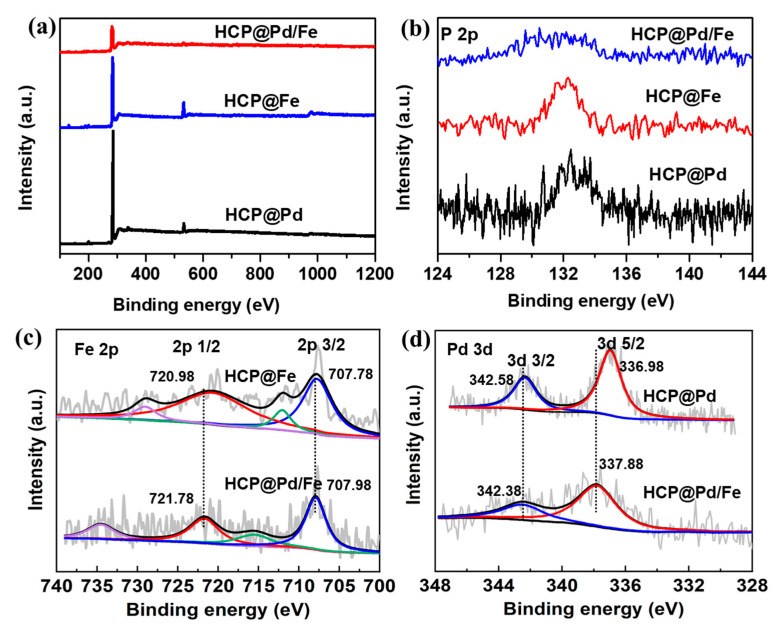
(**a**) XPS total survey spectra of catalysts, (**b**) high-resolution P 2p XPS spectra, (**c**) Fe 2p XPS spectra, and (**d**) Pd 3d XPS spectra of HCP@Pd/Fe, HCP@Fe and HCP@Pd.

X-ray photoelectron spectroscopy (XPS) was utilized to analyze the surface elemental composition and coordination states of different solids. The prepared catalyst exhibited P, C, Pd, Fe, and trace Cl for HCP@Pd/Fe (Figure 4a), consistent with EDS mapping results. The P 2p spectra of HCP@Pd/Fe were identified as an anchor species for immobilized, isolated metals. Upon loading of the bimetal, the binding energy of P 2p in HCP@Pd/Fe slightly shifted toward the lower energy direction (Figure 4b), indicating mutual interactions between Pd and Fe, along with the electron transfer between the metals and coordinated P atoms [40]. The fitted Fe 2p spectra of HCP@Pd/Fe were observed at a binding energy of 707.98 and 721.78 eV, which was 0.2 eV higher than HCP@Fe, suggesting that the chemical state of Fe in the alloy was more active (Figure 4c). For the Pd element, the energy state of Pd 3d 5/2 in HCP@Pd/Fe was found to migrate 0.9 eV to the high-energy region relative to HCP@Pd (Figure 4d). These findings further demonstrate the synergistic interaction among Pd, Fe, and P in the HCP@Pd/Fe alloy catalyst framework, which is conducive to the promotion of the catalytic active sites [41,42].

This current study reports on the catalytic performance of a newly developed PdFe-based catalyst, HCPs@Pd/Fe, in Ullmann coupling reactions. The catalytic activity of HCPs@Pd/Fe was compared to PdCl_2_ and two other Pd-based catalysts, HCP@Pd and HCP@Fe. Results show that HCPs@Pd/Fe had the highest reaction yield (91%, Table 1, entry 1), despite having a lower Pd content compared to PdCl_2_. Furthermore, the catalytic activity of Pd was significantly enhanced by the introduction of Fe, as shown by the low or no yield obtained with HCP@Pd and HCP@Fe (Table 1, entries 2–4). X-ray photoelectron spectroscopy (XPS) characterization revealed that the Pd–Fe synergy and the interaction between the metal and phosphine ligands improved the intrinsic activity of the catalyst and facilitated the activation of the C–Cl bond. The PdFe alloy catalyst, constructed in situ, provides a new design strategy for metal-catalyzed coupling reactions.

**Figure 5:**
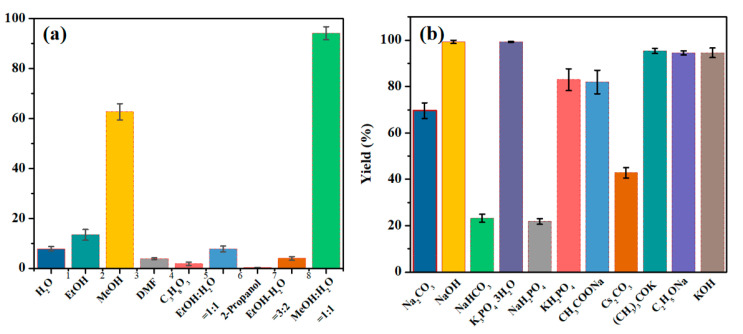
Optimization of Ullmann coupling reaction of chlorobenzene. Reaction condition: chlorobenzene (1 mmol), base (1.5 mmol), solvent (2 mL), catalyst (20 mg), and reaction time (180 min).

Using HCPs@Pd/Fe as the model catalyst, we investigated the effects of different solvents using the Ullmann reaction, and the findings are presented in Figure 5. It was observed that the catalyst was most effective when K_3_PO_4_∙3H_2_O was used as the base in methanol and methanol solution, with yields of 64.5% and 91%, respectively. The yields were significantly lower when solvents such as H_2_O, ethanol, DMF, and propanetriol were used (Figure 5a). Further, we explored the impact of bases in methanol solution (*V*_MeOH_/*V*_H_2_O_ = 1:1, 2 mL), and observed that high activity could be achieved with NaOH, (CH_3_)_3_COK, C_2_H_5_ONa, and KOH as bases (Figure 5b). In the presence of K_3_PO_4_∙3H_2_O, an optimized yield of 99% was achieved at 100 °C (Table S1, entry 3). Additionally, the temperature had a significant effect on the Ullmann coupling reaction, with high temperatures being favorable for the reaction (Table S1).

Using the optimized reaction conditions obtained in the preceding study, we investigated the catalytic performance of several substrates in different coupling biaryls. In Table 2, we report the results of our study on various substituted aryl halides. We found that the catalytic system displayed good to excellent yields for various substituted aryl chlorides. Notably, both electron-donating and -withdrawing groups were tolerated, producing a yield above 80% (Table 2, entries 1–6). However, the reaction yield of *o-*chlorobenzene was lower compared to that of *p-* and *m-*chlorobenzene, under the same reaction conditions, which may be attributed to the steric effect of the group.

### 3.2. Filtration Tests

**Figure 6:**
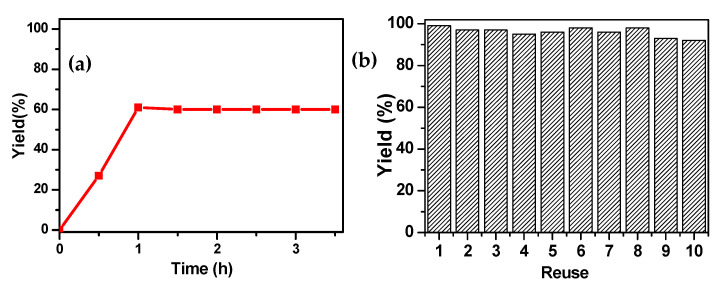
Kinetic curves for the Ullmann coupling reaction of chlorobenzene (**a**), stability testing of the HCP@Pd/Fe catalyzed Ullmann coupling reaction of chlorobenzenes (**b**).

In order to determine whether the Ullmann coupling reaction, using HCPs@Pd/Fe as the catalyst, followed a heterogeneous or homogeneous catalytic pathway, then, hot filtration tests and kinetic studies were conducted [43,44]. After 60 min of reacting, the catalyst was separated from the reaction mixture and only 0.1 ppm palladium was found in the filtrate, indicating minimal leaching of the catalyst. Furthermore, no reaction occurred when a new batch of the substrate was added to the filtrate, indicating that the catalyst was essential for the reaction. Kinetic studies (Figure 6a) showed that the reaction rate did not correlate with the amount of leached Pd species in the solution, indicating that the reaction was intrinsically catalyzed by a heterogeneous catalyst.

To assess the recyclability of the catalyst, the homocoupling reaction of chlorobenzene was carried out under the optimized reaction conditions. The reaction was performed 10 times, and the catalyst was recovered by centrifugation after each reaction, washed with distilled water, and dried at 60 °C. The isolated yields after each run indicated that the catalyst exhibited high stability and recyclability (Figure 6b).

## 4. Conclusions

In this study, a new approach for the synthesis of hyper-crosslinked polymer-coated Pd–Fe alloy catalysts, in situ, was developed. The Pd–Fe alloys exhibited high catalytic activity during the Ullmann reaction of chlorobenzene. The biphenyl yield was observed to be 99% at 80 °C over 120 min and the catalyst could be recycled 10 times without any significant loss of initial catalytic activity. The activity and stability of the bimetallic catalyst were found to be higher than those of the monometallic catalysts, which could be attributed to the interaction between the Pd–Fe metals, thereby promoting electron transfer and enhancing catalytic activity. Furthermore, the polymer with various pores coated on the active site of the metal enhanced the stability of the hyper-crosslinked polymer-coated Pd–Fe alloy catalyst. The advantages of this novel catalyst include high efficiency, easy and rapid separation of the used material, and green and mild reaction conditions, thereby making it a potentially useful and attractive approach for industrial production.

## Figures and Tables

**Table 1:** Ullmann coupling reaction of chlorobenzene with different catalysts.

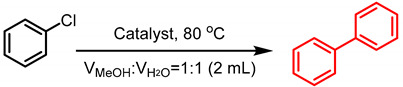					
Entry	Catalyst	Time (min)	Pd (%)	Fe (%)	Yield (%)
1	HCPs@Pd/Fe	180	0.4	0.9	90.6
2	HCP@Pd	180	/	/	35.4
3	PdCl_2_	180	60.0	/	67.3
4	HCP@Fe	180	/	1.2	trace

Reaction conditions: chlorobenzene (1 mmol), K_3_PO_4_∙3H_2_O (1.5 mmol), catalyst (20 mg), and reaction time (180 min).

**Table 2:** Catalytic performance in coupling reaction for substituted chlorobenzene over the HCP@PdFe catalyst.

Entry	Aryl Halides	Biaryls	Time (h)	Yield (%)
1	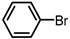	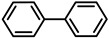	3	99
2	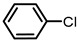	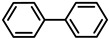	3	99
3	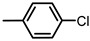	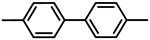	6	93
4	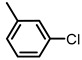	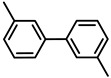	6	80
5	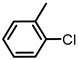	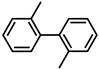	6	70
6	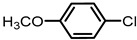	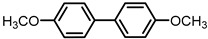	6	90

Ullmann reactions were carried out in air using 1 mmol substituted aryl chloride, 20 mg catalyst, 2 mL solvent, 1.5 mmol K_3_PO_4_∙3H_2_O, and a reaction temperature of 100 °C.

## Data Availability

Not applicable.

## Supplementary Materials

The following supporting information can be downloaded at: https://www.mdpi.com/article/10.3390/polym15122748/s1, Figure S1: The FT-IR spectrum of catalyst HCP@Pd/Fe; Table S1: HCP@Pd/Fe catalyzed Ullmann coupling reaction of different aryl halides.
