# Supplementary material for: Facile Synthesis Hyper-Crosslinked PdFe Bimetallic Polymer as Highly Active Catalyst for Ullmann Coupling Reaction of Chlorobenzene
Source: Polymers (Basel). 2023 Jun 20;15(12):2748. doi: 10.3390/polym15122748 (PMC10304214; doi:10.3390/polym15122748)
Supplement: Supplementary file 1 [file polymers-15-02748-s001.zip › polymers-2423840-supplementary.pdf]

Supporting Information

Facile Synthesis Hyper-Crosslinked PdFe Bimetallic Polymer as Highly Active Catalyst for Ullmann Coupling Reaction of Chlorobenzene

Cheng Tang [1,2,†], Wenwen Yang [1,†], Zhijuan Zou [1,*], Fang Liao [1], Chunmei Zeng [1] and Kunpeng Song [1,*]

[1] Chemical Synthesis and Pollution Control Key Laboratory of Sichuan Province,
College of Chemistry and Chemical Engineering, China West Normal University, Shida Road,
Nanchong 637009, China; 20200043@sasu.edu.cn (C.T.); wenwenyang85@163.com (W.Y.); liaofang407@163.com (F.L.); meizeng@163.com (C.Z.)

[2] Key Laboratory of Low-cost Rural Environmental Treatment Technology, Sichuan University of Arts and Science, Education Department of Sichuan Province, Dazhou 635000, China

* Correspondence: zouzhijuan121@163.com (Z.Z.); song19880405@126.com (K.S.)

† The authors contributed equally to this paper.

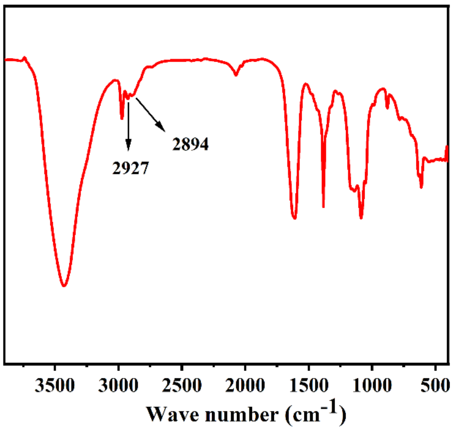

Figure S1. The FT-IR spectrum of catalyst HCP@Pd/Fe.

Table S1 HCP@Pd/Fe catalyzed Ullmann coupling reaction of different aryl halides.

**Entry**	**Substrate1**	**Base**	**Solvent (2ml)**	**T(°C)**	**Yield (%)**
1	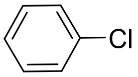	$K_3PO_4·3H_2O$	CH_3OH: H_2O=1:1	80	91
2	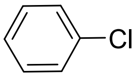	$K_3PO_4·3H_2O$	CH_3OH: H_2O=1:1	90	97.7
3	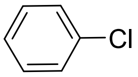	$K_3PO_4·3H_2O$	CH_3OH: H_2O=1:1	100	100
4	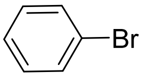	$K_3PO_4·3H_2O$	CH_3OH: H_2O=1:1	100	99.1
5	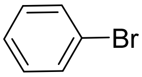	$K_3PO_4·3H_2O$	CH_3OH: H_2O=1:1	80	96.3
6	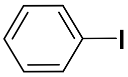	$K_3PO_4·3H_2O$	CH_3OH: H_2O=1:1	80	60

Reaction conditions: aryl halide (1 mmol), $K_3PO_4·3H_2O$ (1.5 mmol), V_{MeOH}/V_{H2O}=1:1 (2 ml), catalyst (20 mg), reaction time 180 min.
